# Abdominal Wall Hernias: An Epidemiological Profile and Surgical Experience from a Rural Medical College in Central India

**DOI:** 10.1055/s-0040-1722744

**Published:** 2021-03-11

**Authors:** Bharati Pandya, Tanweerul Huda, Dilip Gupta, Bhupendra Mehra, Ravinder Narang

**Affiliations:** 1 Department of General Surgery, AIIMS, Bhopal, India; 2 Department of General Surgery, L. N. Medical College, Bhopal, India; 3 Department of Surgery, Mahatma Gandhi Institute of Medical Sciences, Wardha, India

**Keywords:** abdominal wall, hernias, hernia surgery, rural setup, India

## Abstract

**Background** Abdominal wall hernia is a common surgical entity worldwide with groin hernias having the most common presentation among them. They are a cause of morbidity and mortality if not addressed in time. A variety of surgical methods are available for the repair of hernias. The tension-free repair using synthetic mesh has the least recurrence and is the most accepted.

**Aim** To describe the surgical burden and clinical profile of abdominal wall hernias as well as experiences in their management in a rural setup.

**Methods** This was a retrospective observational study of all the cases of abdominal wall hernias presenting to various surgical divisions of Mahatma Gandhi Institute of Medical Sciences, Sevagram, during a two-year period from December 2011 to November 2013. Relevant details were collected from the hospital information statistics and patient file records and analysis of obtained data was done.

**Result** A total of 910 out of 90,056 surgical outpatients (10.10%) seen during this period had abdominal wall hernias; 816 (89.67%) got operated. A total of 163 (20%) of 816 were operated in an emergency. Groin hernias were the most common 653 (80%), followed by incisional 82 (10%), umbilical and paraumbilical 41 (5%), epigastric 33 (4%), and rarer hernias in 8 (1%). Of 816 operations, 24 (2.9%) had recurrent hernias and 83 (10.17%) were pediatric patients. Male to female ratio was 9:1 in adults and 4:1 in children. The median age among adults was 49 years (range: 14–95 years), and among the pediatric age group, it was 7 years (range: 3 months–14 years). The majority of the adult patients were from a low-income group and presented more than 2 years after symptoms appeared. Comorbid conditions encountered were hypertension in 212 (26%), diabetes in 155 (19%), chronic airway disorders in 449 (55%), cardiac problems in 163 (20%), obesity in 10 (1.2%), and chronic renal failure and liver disorder in 82 (1%). Predisposing factors in the majority of the patients were chronic cough 449 (55%), prostatic problems in 187 (23%), chronic constipation in 163 (20%), previous surgeries in 82 (10%), obesity in 10 (1.2%), and ascites in 9 (0.1%). Hernia surgery was performed laparoscopically in 51 (6.25%) patients. Simultaneous other surgeries were performed in 130 (16%) patients. Mortality occurred in 2 (0.24%) patients operated in emergency, and chief morbidity was due to wound infection in 25 (3%) and chronic pain in 30 (3.9%) patients.

**Conclusion** Abdominal wall hernias are common clinical entities. Although the pattern of presentation and management is similar, the challenges faced in a rural setup are due to ignorance, social inhibitions, and financial restraints, leading to delayed presentations which increase their morbidity and mortality. Health programs and surveys to increase awareness in rural areas as well as cutting down on expenses could help these patients.

An abdominal wall hernia is an abnormal protrusion of a peritoneal-lined sac through the musculo-aponeurotic covering of the abdomen. 1 The most common variety is groin hernias, of which inguinal hernias (direct and indirect) are far more common than femoral hernias. Hernias of the abdominal wall are quite common, having a prevalence of 1.7% for all ages and 4% for those older than 45 years. 2 Hernias of the inguinal region account for 75% of abdominal wall hernias, with a lifetime chance of 27% in males and 3% in females. 2 More than 20 million hernias are estimated to be repaired all over the world every year. The other varieties are umbilical, paraumbilical, epigastric, incisional, and rarer ones like Spigelian and traumatic hernias. The inguinal hernia has a male preponderance, while femoral hernias are more common in females. Common in the pediatric age group is indirect inguinal and umbilical hernias. Symptomatology is usually suggestive, and clinical examination is the mainstay of diagnosis. However, some hernias may need radioimaging for diagnosis. Mesh repair is the preferred method of repair due to decreased recurrence rates, but herniorrhaphy is a cost-effective method of choice for many patients. Herniotomy and Mayo's repair are the procedures of choice in pediatric patients. Laparoscopic surgery has become more common due to low postoperative morbidity and early return to work but is not very cost-effective in rural setups.

The problem with developing countries, especially in their rural population, is medical ignorance, cost restraints, and social inhibitions. Delayed presentations add to postoperative morbidity and mortality. The aims are to train the residents in surgical units and to perform the most cost-effective surgeries with minimum morbidity, ensuring early return to work for these patients. Simultaneous efforts are required to conduct area-wise surveys, educating the masses, and cutting down on expenses, thereby making an early diagnosis and providing the best techniques available to these strata of society.

Our aims in this study were to assess the clinical burden and profile of hernia patients in a rural medical college and to describe our experience in managing them.

## Methodology

This rural district has an area of approximately 6,310 km ^2^ and a population of around 15 lakhs with Asian ethnicity. As much as 76% of the total population is rural, with 42% of the population living below the poverty line. There are only two teaching medical college hospitals (tertiary care centers) serving this area. This was a retrospective study conducted at our rural medical college at Mahatma Gandhi Institute of Medical Sciences, which is a tertiary medical care center situated in the central part of India. It is a trust-run hospital with aid being received from the government. It is based on self-paying with minimum charges due to government and state funding.

All the patients of external abdominal wall hernias who attended the different surgical sections from December 1, 2011, to December 1, 2013, were included in the study. A proforma was designed and relevant details were collected from the hospital information statistics and clinical records of the patients seen during these 2 years. Surgery is performed by general surgeons along with surgical officers who are also well-versed in laparoscopic surgeries. Due to a lack of pediatric surgeons at this setup, the pediatric hernias are also managed by general surgeons. Data included the sociodemographic information, presentation details, comorbid conditions, choice of anesthesia and surgical procedures along with their outcomes and follow-up details,

The obtained data were analyzed using frequency distribution, percentages, range, mean, tables, and charts to obtain the results. Statistical tests were performed to relate the relevance as was felt necessary.

## Results

The study was performed by retrospective analysis of data on all anterior abdominal wall hernias in our hospital, a tertiary care hospital with an attached medical college in a rural setup, which is the first of its kind in the country.

**Fig. 1:**
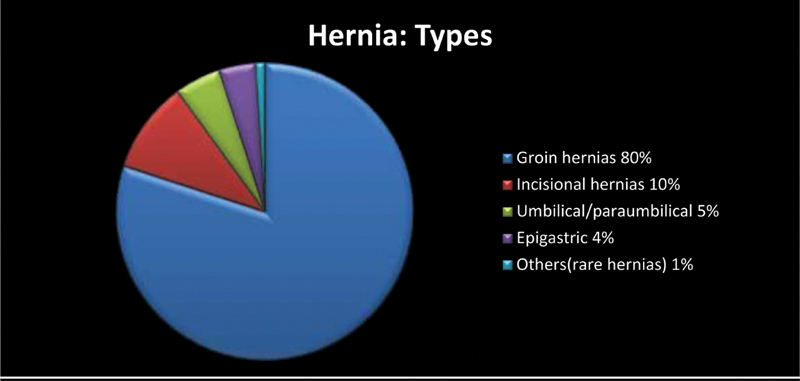
Different types of hernias.

The total surgical outpatient attendance during this period of two years was of 90,056. A total of 910 (10.10%) of these patients had anterior abdominal wall hernias. A total of 816 patients presented themselves for surgery. Groin hernias accounted for 80% of the lot, while incisional hernias (10%), umbilical/paraumbilical (5%), epigastric (4%), and rare hernias (1%) mad up the rest ( Fig. 1 ).

**Fig. 2:**
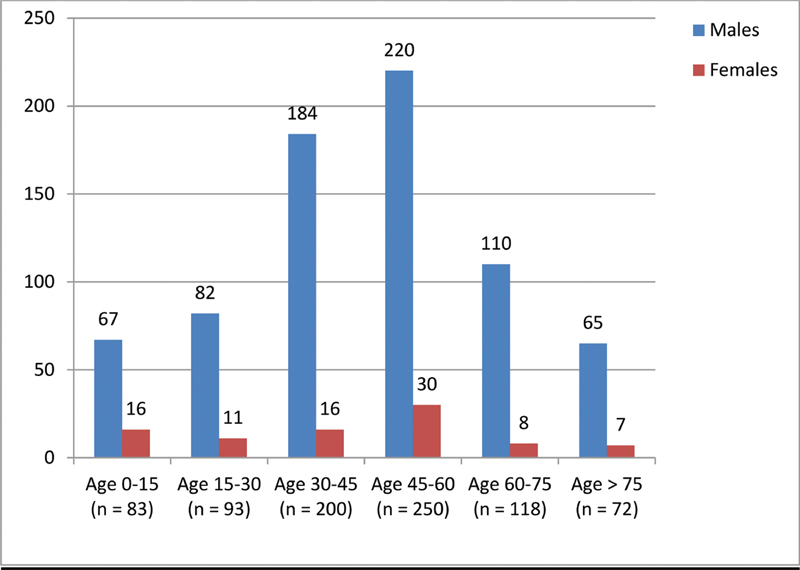
Age-wise frequency of presentation.

The male (728) to female (88) ratio was 9:1 in adults and 4:1 (67:16) in children. The median age among adults was 49 years (range: 14–95 years), and among the pediatric age group, it was 7 years (range: 3 months–14 years) ( Fig. 2 ).

Predisposing factors in a majority of the patients were chronic cough 449 (55%), prostatic problems in 187 (23%), chronic constipation in 163 (20%), previous surgeries in 82 (10%), obesity in 10 (1.2%), and ascites in 9 (1.1%) ( Table 1 ). In females, additional factors of multiparity in 50 (6.1%) and transabdominal gynecological surgeries in 32 (4.0%) patients were observed. In pediatric and young adults, the preperitoneal sac in 79 (89%), excessive crying and weak musculature attributed to 21 (25%), and chronic respiratory infections in 19 (23%) were the predisposing factors discernable.

**Table 1:** Predisposing factors and co-morbid conditions

I. Predisposing Factors	Male (728)	Female (88)	Total ( *n* = 816)	%age
Excessive crying (in children)	17	4	21/83	25
Chronic resp. infections (in children)	15	4	19/83	23
Pre-peritoneal sac	203	15	218	26.7
Strenuous exercises	80	2	82	10
Chronic cough/smoking	400	49	449	55
Bladder outlet obstruction	186	2	188	23
Chronic constipation	81	82	163	20
Previous surgeries	48	34	82	10
Obesity	2	8	10	1.2
Ascites	3	6	9	1.1
Multiparity	0	50	50	6.1
**II. Comorbidity**	**Male**	**Female**	***n*** ** = 816**	**%age**
Hypertension	152	60	212	26
Diabetes	85	70	155	19
Chronic airway disorders	400	49	449	55
Cardiac problems	108	55	163	20
Obesity	4	6	10	1.2
Chronic renal disorders	2	1	3	0.36
Chronic liver disorders	4	1	5	0.61

Comorbid conditions encountered were hypertension in 212 (26%), diabetes in 155 (19%), chronic airway disorders in 449 (55%) cardiac problems in 163 (20%), obesity in 10 (1.2%), chronic renal failure in 3 (0.36%), and liver disorder in 5 (0.61%) ( Table 1 ).

Of 816 operations, 24 (2.9%) had recurrent hernias and 83 (10.17%) were pediatric patients, mostly congenital inguinal and epigastric. Most of our study patients had normal build and only 10 (1.2%) cases of obesity were recorded. The bilateral hernia was seen only in 56 (6.56%) patients. As much as 73% of the hernias were acquired, of which 74% were incomplete and approximately 84% were indirect. Cough impulse and reducibility were seen in approximately 75% of the patients with hernias.

Surgeries performed were subject to the type of hernia. Open Lichtenstein's repair was performed in the majority of inguinal hernias, followed by Bassini's repair. Open Meshplasty or Lap Meshplasty was the surgery of choice for umbilical, epigastric, incisional, and recurrent hernias, depending on the affordability of the patient. Pediatric inguinal hernias underwent herniotomy. Pediatric and few adult hernias were repaired with Mayo's procedure. Mesh was avoided in children. Laparoscopic surgery was performed in only 51 (6.25%) patients ( Table 2 ).

**Table 2:** Types of hernia, symptomatology, surgeries performed, and their complications

S. No.	Types of hernia	*n* = 816	Clinical features	Method of repair	Complications *n* = 66 (8.0% of patients)
**1.**	Direct inguinal hernia	*n* = 349 (42.8%)	Elderly Reducible inguinal swelling	Lap-Hernioplasty Lichtenstein's repair	1. Hematoma/seroma 10 (1.2%) 2. Wound infection and dehiscence 20 (2.4%) 3. Scrotal Hematoma 6 (0.7%) 4. Mesh extrusion/ removal 2 (0.24%) 5. Nerve entrapment & pain 20 (2.4%) 6. Recurrence 6 (0.73%) 7. Mortality 2 (0.24%)
**2.**	Indirect inguinal hernia	*n* = 252 (30.9%)	Children, young adults > elderly Reducible/partially reducible irreducible Pain	Bassini's repair Lichtenstein's repair Lap hernioplasty	
**3.**	Femoral hernia	*n* = 4 (0.4%)	Irreducible Pain Obstructed	Lotheissen's repair Lap-hernioplasty	
**4.**	Epigastric hernia	*n* = 33 (4%)	Epigastric bulge Irreducible/partially reducible Pain	Anatomical repair Meshplasty	
**5.**	Umbilical hernia	*n* = 25 (3%)	Bulge Reducible/irreducible Obstructed	Meshplasty Mayo's repair	
**6.**	Paraumbilical hernia	*n* = 16 (2%)	Bulge Irreducible/reducible	Meshplasty Mayo's repair	
**7.**	Incisional hernia	*n* = 82 (10%)	Previous surgery Bulge Reducible/partially reducible Irreducible Pain	Meshplasty Lap-hernioplasty	
**8.**	Spigelian hernia	*n* = 3 (0.35%)	Bulge Reducible	Meshplasty	
**9.**	Traumatic hernia	*n* = 5 (0.65%)	Bulge H/o trauma Occasional pain	Meshplasty	

Morbidity was mainly due to wound infection in 20 (2.4%). Long-term morbidity was due to chronic pain in 20 (2.4%) patients. Recurrence was documented only in 6 (0.7%) cases who reported back within a year ( Table 2 ). The exact figures could not be quoted as long-term follow-up was not feasible.

A total of 163 (20%) patients were operated in emergency and had presented with irreducibility and pain. Strangulation was present in 5 (0.61%) patients requiring resection anastomosis. Mortality occurred in 2 (0.24%) patients operated in an emergency; one had a perforation of a gangrenous segment in the scrotum, presenting very late with Fournier's gangrene, and the second, who had cirrhosis with a strangulated umbilical hernia and skin excoriations, succumbed postoperatively.

Other surgeries were performed simultaneously in 130 (16%) patients, with the most common being opposite side hernia ( Table 3 ).

**Table 3:** Simultaneous surgeries performed

S. No	Simultaneous surgeries performed	*n* = 130 (16.0%)	%	Diagnosis	Herniotomy/herniorrhaphy/hernioplasty PLUS
**1.**	Hydrocele	18	13.8	Clinical/USG proven	Eversion of sac
**2.**	Undescended testis	7	5.4	Clinical/USG proven	Orchidopexy/orchidectomy
**3.**	Contralateral hernia	30	23	Examination findings	B/L hernia repair
**4.**	Inguinal + other hernias	17	13	Examination findings	Hernioplasty/Mayo's repair
**5.**	Bladder outlet obstruction	20	15.5	Urinary complaints USG findings	TURP/OIU
**6.**	Hemorrhoids	10	7.7	Bleeding P/R Proctoscopy	Hemorrhoidectomy
**7.**	Bowel gangrene	5	3.8	S/S of strangulation	Resection and anastomosis
**8.**	Cholelithiasis	5	3.8	USG proven	Laparoscopic cholecystectomy
**9.**	Appendix in sac	4	3.2	Operative finding	Appendicectomy
**10.**	Gynecological procedures	14	10.8	Planned gynecological procedures	Gynecological surgeries

Abbreviations: OIU, optical internal urethrotomy; TURP, transurethral resection of the prostrate.

## Discussion

This retrospective, observational study was done to look for the various etiological factors and clinical presentations of different varieties of abdominal wall hernia. It was also a complete profile study to learn the effect of various comorbidities, surgical options, and outcomes feasible in a rural setup. The study is from a tertiary care center located in rural India which has also begun with laparoscopic procedures for hernial repair.

In infants, hernias are attributed to preformed sacs, and it has been said that inguinal hernia is a disease of infants due to defect in the inguinal canal by some, while others have found a higher incidence of inguinal hernia in higher age groups. Our study demonstrated that inguinal hernia was seen with an increase in age but equally true is that there were many pediatric patients with congenital inguinal hernia also. Literature quotes the mean age of inguinal hernia to be 40 to 60 years. 3 Our experience was similar, and this age group leads to an economic burden on account of the productive age group being affected. In all age groups, males were more affected. Literature quotes a ratio of 20:1 in males as compared with females. However, femoral hernias, epigastric hernias, and incisional hernias are more common in females, who delay their presentation due to a variety of reasons. In the Indian rural population, the main reasons are their family responsibilities and inability to approach hospital care due to social inhibitions, fear of operative intervention, and the tendency to conceal their medical issues. Factors responsible in males are strenuous activities, chronic cough, and preformed sacs. 3 4 5 Even males tend to present late due to their fear of surgery and loss at work, causing financial burden on the family. These factors were comparable to other developing countries. 6 7 8

The most common surgical procedure opted for was Lichtenstein's mesh repair technique, which is the simplest and has the advantage of being an open, tension-free repair with the least rates of recurrence. 3 9 10 For children, the choice was herniotomy with minimal postoperative complications. We performed laparoscopic repairs in 51 (6.25%) patients at our setup. The reason behind this choice is an early return to work, faster recovery, and decreased morbidities like nerve entrapment. The cost of the procedure was a cause of restraint in our patients; also, the time taken is more for laparoscopic procedures as compared with open and it matters, as the resultant time of anesthesia and affection of the time management of operating theater happens to be a major concern.

In our observational study, the complication rates were 8.0%, which is relatively less compared with 4.2% to as high as 12.4% as quoted by other series in the literature. 8 Our study had 86.8% of cases performed electively as compared with 61.5% cases in other series, and this could have been a probable factor for low complications. 8 Emergency surgeries performed on complicated hernias led to the mortality quoted, due to perforation and strangulation of the incarcerated bowel loop, thus proving the fact that neglect and late presentation results in dismal outcomes. The coexistent morbidities also add to the overall morbidity, as patients develop respiratory and cardiac complications that are difficult to manage postoperatively. Additional surgeries performed in our study did not add to either morbidity or mortality yet saved the patient the trauma of a second procedure, and we would recommend it unless it has a direct bearing on the healing process, like active infection and chances of contamination, which may then be contraindications to performing them simultaneously with a hernia repair.

## Conclusion

Abdominal wall hernias are a common entity and account for approximately 10% of our total outpatient attendance. Most of the patients in our rural setting presented late, more than 2 years after developing the hernia and often with complications. Proper health awareness, especially among the rural population and females, is what is important and is advocated. Mesh repair is the standard procedure applicable to most of the population. A laparoscopic hernioplasty is best suited to these population groups, as it results in a lesser hospital stay and early return to work. Laparoscopy has a longer learning curve however and hence requires to be customized as per the need of the population, and reduction in its financial implications could be beneficial.
